# Impact of Prolonged Exposure to a Slippery Surface on Postural Stability

**DOI:** 10.3390/ijerph18052214

**Published:** 2021-02-24

**Authors:** Sachini N. K. Kodithuwakku Arachchige, Harish Chander, Alana J. Turner, Adam C. Knight

**Affiliations:** Neuromechanics Laboratory, Department of Kinesiology, Mississippi State University, Starkville, MS 39762, USA; hchander@colled.msstate.edu (H.C.); ajt188@msstate.edu (A.J.T.); aknight@colled.msstate.edu (A.C.K.)

**Keywords:** extended, chronic, dry, balance, load-carrying, ergonomics, occupational falls

## Abstract

Falls are extremely common in occupational settings. Intrinsic factors such as overexertion and extrinsic factors such as the supporting surface are causative factors of falls. The impact of prolonged exposure to a slippery surface on postural stability has not been previously studied. The purpose of the study was to analyze the effect of extended exposure to a dry and a slippery surface on postural stability. Eighteen males (age: 21.17 ± 3.38 years; height: 1.77 ± 0.08 m; mass: 89.81 ± 14.23 kg) were recruited and subjected to one-hour walking on a dry surface and a slippery surface on two different days. Participants’ balance was assessed using a force platform in stable and unstable conditions at 0, 30, and 60 min. Postural sway variables were analyzed using a 2 (surface) × 3 (time) repeated-measures ANOVA. Significant time main effects were observed in the stable condition with greater balance decrements at 30 and 60 min. Greater balance decrements were observed on the slippery surface compared to the dry surface in the unstable condition. The balance decrements can be attributed to overexertion due to the physiological workload of prolonged walking and to the potential gait modifications due to walking on the slippery surface.

## 1. Introduction

In the year 2017, a total of 887 fatal occupational falls and 227,760 nonfatal occupational falls were reported by the U.S. Bureau of Labor Statistics [1,2]. These lethal falls accounted for 17% of the total occupational deaths and were the highest incidence of fall-related occupational deaths recorded in the Census of Fatal Occupational Injuries (CFOI) in 26 years [1]. The prevalence of occupational falls is higher among construction and manufacturing workers, roofers, and agricultural workers [2,3]. Work-related falls have been increasing over the years, threatening workers’ health, productivity, and quality of life [2]. Medical expenses, compensation, and replacement workers increase the economic burden on the workplace. Annually, more than $70 billion is allocated to the management of occupational falls in the United States of America [4]. Slips and trips leading to loss of balance have been identified as one of the major causative factors for falls in the workplace [2,5] and have been attributed to 14.8% (33,720 falls) of all occupational injuries in 2017 [2].

A fall occurs upon the inability to recover from an induced loss of balance [6]. Falls occur due to extrinsic (environmental) and intrinsic (human) factors. The nature of the supporting surface is one of the crucial extrinsic factors [7], and muscular fatigue is one of the major intrinsic factors for such falls [8]. These causative factors act as an external or internal perturbation, challenging the ability of the postural control system to maintain a stable center of mass (COM) within their base of support (BOS) [9]. In the realm of biomechanics, many studies have been conducted to observe the impact of standing and walking surfaces on postural stability. Depending on the occupation, the nature of the supporting surface varies, such as stable/unstable, flat/inclined, contaminated/clean, and slippery/dry. Since the workers are standing or walking on the supporting surface throughout the working day, it has a major influence on maintaining upright balance. Working on an unstable, inclined, contaminated, or slippery surface is more challenging compared to working on a stable, flat, or clean surface. In studying the biomechanics of slips, the impact of acute exposure to a slippery surface on postural stability has been examined in healthy individuals [10], the geriatric population [11], and in professional occupational groups such as firefighters [12]. Some groups have studied the impact of different footwear, expected and unexpected perturbations, and load carriage on slip biomechanics [12,13,14,15,16]. Further, static postural stability, joint kinematics, muscle activity, slip velocity and slip severity have been observed [5,11,12,16,17]. Due to the abundance of slip-related falls at the workplace, most of these studies were focused on the ergonomic population [15,16,18]. Although the prolonged exposure to a slippery surface is commonly seen in certain occupations such as construction workers, manufacturers, and restaurant kitchen workers, to date, the effects of chronic exposure to a slippery surface on postural stability have not been studied. However, extended exposure to a dry surface has been studied, referring to different occupational populations such as roofers, railroad workers, manufacturing, and assembly line workers on flat surface [12,13] and on inclined surfaces [14,15]. In a study done by Chander et al., fourteen young, healthy males were subjected to walking for four hours on a dry surface, and their static balance was assessed at every 30-min interval. They observed balance decrements over time, attributed to prolonged walking causing fatigue in the participants [13]. In addition, overexertion has been identified as a major contributing factor of slip-induced falls [5]. Therefore, a higher incidence of postural instability following extended exposure to a slippery surface could be speculated.

Load-carriage has been identified as a causative factor of fall-related occupational injuries [18] Postural instability has been observed even during static stance while holding an external load [19]. Heller et al. observed young healthy females’ static stability with and without an 18.1 kg external load during quiet standing for 30 s. They have observed significantly higher postural sway while holding the external load than without holding the load. Furthermore, the impact of different types of occupational load-carrying tasks on balance has been studied [20,21]. Chander et al. subjected a group of young, healthy males to treadmill walking while carrying a 16 kg rucksack until fatigue, simulating a military-type workload. Balance decrements were observed following the load-carrying task, attributed to overexertion and muscle fatigue [22]. Usually, anterior load carrying is commonly seen compared to posterior or lateral load-carriage in occupational settings. However, anterior load-carriage has not been vastly studied before. Robert et al. studied 29 individuals with different magnitudes (no-load, load equals to 0%, 5%, and 10% of body mass) of anterior load-carriage and observed higher center of pressure (COP) displacements with greater load, suggesting the role of the quantity of external load on balance decrements [23].

Moreover, load-carriage and fatigue are reported to influence the spatiotemporal parameters of gait and lower extremity joint kinematics [23,24,25,26,27]. Improper carriage of loads, excessive amounts of loads, and load-carriage for extended durations can cause undue fatigue and overexertion. With load carrying and overexertion, increased stride length and decreased stride rate [23], higher stride rate and shorter stride length, decreased pelvic rotation [27], decreased stance time [25], increased step length, stride length, cadence, ankle dorsiflexion, knee flexion and hip flexion [26] have been reported. Thus, it is apparent that the nature of the standing surface, load-carriage, and overexertion could impact postural stability. Therefore, the purpose of this study was to analyze the impact of extended exposure to a dry surface and a slippery surface during a simulated work task on static postural stability in young, healthy males. The study was designed to investigate two aims. The primary aim was to assess the impact of the surface on postural stability, and the secondary aim was to assess the impact of time on postural stability. It was hypothesized that greater balance decrements would be observed with increased walking time and on the slippery surface.

## 2. Materials and Methods

### 2.1. Participants

Eighteen healthy males (age: 21.17 ± 3.38 years; height: 1.77 ± 0.08 m; mass: 89.81 ± 14.23 kg; body mass index: 28.83 ± 4.40 kgm^2^) with no history of musculoskeletal, visual, vestibular, neurological, or cardiovascular disorders were recruited. Only recreationally active individuals were selected for participation (a minimum of 3–4 days per week (150min) of aerobic exercise and a minimum of 2 days per week of resistance training for at least the last 3 months [28]). 

### 2.2. Study Design 

The study was approved by the Mississippi State University (MSU) Institutional Review Board (IRB-19-135) and was carried out entirely in the MSU Neuromechanics Laboratory. The study followed a repeated measures design with a counterbalanced surface assignment (dry and slippery surface).

### 2.3. Procedures

Upon arrival for the initial familiarization day, the testing protocol was described to the participants, and consent was obtained. To identify any health-related risk factors, participants answered a Physical Activity Readiness Questionnaire (PAR-Q). Following taking the anthropometric measurements, participants were randomly selected to the “dry” or “slippery” group. The participants were exposed to a standard backpack type fall-arrest harness that meets Occupational Safety and Health Administration (OSHA) guidelines. In addition, the allocation of slip-resistant footwear to the participants was done on the same day. Treadsafe^®^ slip-resistant footwear (Treadsafe, Durban, South Africa) was used in the study due to its ability to provide slip resistance on wet and oily surfaces. Participants were advised to wear the slip-resistant shoes and the fall-arrest harness on both days (on dry and slippery surfaces) to maintain the study’s consistency. After answering any questions participants had, the familiarization day ended.

On the next convenient day, participants arrived at the lab for day one of testing. Participants who were categorized into the “dry” group were tested on the dry surface, and the participants who were categorized into the “slippery” group were tested on the slippery surface on day one of testing. Upon preparing the participants with slip-resistant shoes and the fall-arrest harness, their static postural stability was assessed at 0 min using a force platform (AMTI AccuGait, Watertown, MA, USA). Balance data was collected for three trials, each on stable and unstable (foam pad) surfaces in the eyes opened (EO) condition for 20 s. The participants were advised to stand motionless on the force platform, with arms at their sides, gaze fixed, without speaking. 

After the 0-min static balance assessment, the participants were directed to the walking path (1 m × 3.66 m). A clean floor was maintained for the testing on the dry surface. For testing on the slippery surface, the floor was prepared with evenly distributed industrial vegetable-based glycerol and water mixture in the ratio of 3:1 [12]. The participants were advised to walk at a self-selected pace for one hour along the walking path while carrying a box (0.32 × 0.24 × 0.22 m), which weighed 10% of their body mass (8.98 ± 1.42 kg) [29]. Two tables were placed at each end of the walking path. The participants were asked to set and lift the box occasionally (a total of 30 lifts and 30 set downs within the hour) to simulate a lifting and carrying task in the occupational settings. At 30 min of walking time, the walking was paused briefly, and the participants were directed to the force platform beside the walkway to assess static balance. The static balance testing session took less than 5 min, and the stopwatch was paused during the balance assessments and was restarted once the participants resumed walking. At 60 min of walking time, the static balance was assessed, which marked the end of day one of testing. The same protocol was repeated for the other surface (dry or slippery) following a minimum of 72-h rest period [22].

### 2.4. Statistical Analysis

Postural sway variables of interest were center of pressure (COP)-X average (cm), COP-Y average (cm), average displacement along *X*-axis (cm), average displacement along *Y*-axis (cm), and 95% ellipse area (cm^2^). The *X*-axis represented the anteroposterior direction, while the *Y*-axis represented the medial-lateral direction. Higher values for the sway variables indicate greater balance decrements. The average of three trials for each sway variable was analyzed using a two-way [time (0 min, 30 min, 60 min) and surface (dry, slippery] repeated measures analysis of variance (ANOVA) for each condition. Any significant main effect was further analyzed with post hoc pairwise comparisons using the Bonferroni correction factor. All statistical analyses were done using SPSS statistical software ver. 27 (IBM, Armonk, NY, USA) with a *p* value set a priori at 0.05. 

## 3. Results

### 3.1. Time of Exposure and Static Postural Stability

**Figure 1:**
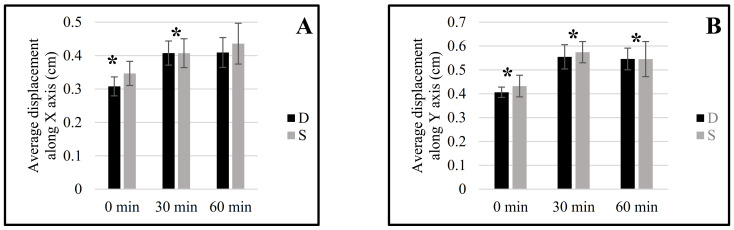

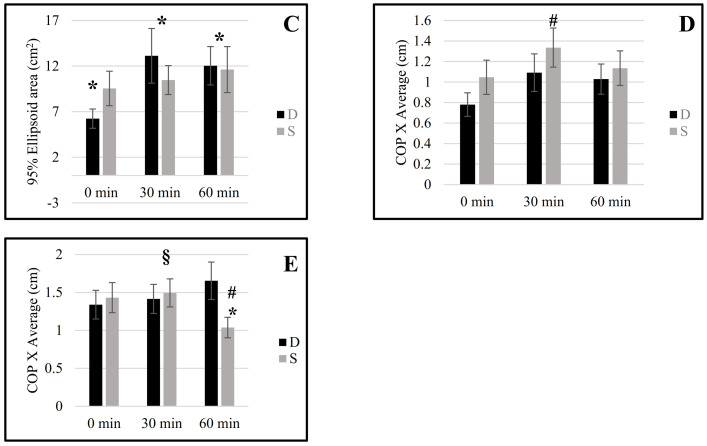
(**A**) Average displacement along the anteroposterior axis (*X*-axis) in the stable condition. * represent significant time differences. (**B**) Average displacement along *Y*-axis in the stable condition. * represent significant time differences. (**C**) 95% ellipsoid area in the stable condition. * represent significant time differences. (**D**) COP in the anteroposterior axis (COP-X average) in the unstable condition. # represents significant surface differences. (**E**) COP in the anteroposterior axis (COP-X average) in the stable condition. § represent significant time × surface interaction. # represents a significant difference between surfaces. * represents a significant difference from 0min and 30min. Bars represent the standard error. D: dry surface; S: slippery surface.

The two-way repeated-measures ANOVA revealed a significant time main effect in the stable condition (Table 1 and Table 2). A significant time main effect for average displacement along the anteroposterior direction (*F* (2, 34) = 4.545; *p* = 0.018; *ηp^2^* = 0.211) was observed. Further analysis with post-hoc comparisons (Bonferroni) revealed significantly greater displacement along the anteroposterior direction at 30 min compared to 0 min (*p* = 0.017) (Figure 1A). Similarly, there was a significant time main effect for average displacement along the medial lateral direction (*F* (2, 34) = 11.439; *p* < 0.001; *ηp^2^* = 0.402) in the stable condition. Further analysis with Bonferroni post-hoc comparisons revealed significantly greater displacement along the medial lateral direction at 30 min (*p* = 0.001) and at 60 min compared to 0 min (*p* = 0.004) (Figure 1B). In addition, a significant time main effect for 95% ellipsoid area was observed (*F* (2, 34) = 5.415; *p* = 0.009; *ηp^2^* = 0.242) in the stable condition. Further analysis with post-hoc comparisons revealed significantly greater 95% ellipsoid area at 30 min (*p* = 0.011) and at 60 min compared to 0 min (*p* = 0.030) (Figure 1C).

### 3.2. Nature of the Standing Surface and Static Postural Stability

The analysis revealed a significant surface main effect for COP in the anteroposterior axis (COP-X average) (*F* (1, 17) = 4.498; *p* = 0.049; *ηp^2^* = 0.209) in the unstable condition (Table 1 and Table 2). The descriptive statistics demonstrated significantly greater COP-X average on the slippery surface (*M* = 1.17; *SD* = 0.53) compared to the dry surface (*M* = 0.97; *SD* = 0.41) (Figure 1D and Table 1).

In addition, a significant time × surface interaction was observed in the stable condition for COP-X average (*F* (2, 34) = 4.459; *p* = 0.019; *ηp^2^* = 0.208) (Figure 1E). The significant interaction was followed up with simple effects analysis. For the slippery surface, COP-X at 60 min was significantly lower compared to 0 min and 30 min. For the 60th minute, COP-X for the slippery surface was significantly lower compared to the dry surface.

## 4. Discussion

The purpose of this study was to analyze the impact of extended exposure to a dry surface and a slippery surface during a simulated work task on postural stability in young, healthy males. Five postural sway variables were considered to analyze the static balance on two different testing conditions (stable and unstable). Higher values for postural sway variables represented greater COP excursions indicating greater balance decrements. Results from the study showed significant balance differences with time and the surface. Overall, higher balance decrements were observed over time and on the slippery surface.

### 4.1. Physiological Workload and Prolonged Walking on Postural Stability 

In the present study, greater balance decrements were observed at 30 min and 60 min compared to 0 min in the stable condition. These changes were observed along the anteroposterior direction (average displacement along *X*-axis and 95% ellipsoid area) and medial-lateral direction (average displacement along *Y*-axis and 95% ellipsoid area). The observed results could be attributed to the overexertion (fatigue) caused by the load carrying and walking for a prolonged duration.

Load carriage resulting in fatigue and balance decrements has been widely studied before. Load carriage is shown to cause alterations to postural sway, ground reaction force, spatiotemporal parameters of the gait, and joint kinematics leading to poor postural stability [22,23,29,30]. The balance decrements were shown to be increased with the magnitude of the load and the carrying time [23]. Carrying an external load displaces the body’s COM from its usual position. The present study included an anterior load-carrying task, shifting the participants’ COM anteriorly. Therefore, the trunk leans posteriorly in order to maintain the COM within the BOS [31]. Further, the use of both hands to carry the load prevents the compensatory movements of the arms during an event of imbalance. Anterior load carriage is shown to increase the anteroposterior postural sway previously [23,32], and the current study demonstrates similar findings. Furthermore, the study included a 10% load carriage task. The previous researchers who have incorporated 10% of the participants’ body weight have shown similar findings to the current study [23,29,32]. There is still a dearth of literature in anterior load-carriage causing postural decrements in ergonomic settings. This study would contribute to the existing minimal literature in this area of ergonomics. 

Moreover, carrying a load itself induces fatigue. Physiological workload and fatigue- causing balance decrements have been observed previously [9,13,14,30]. Fatigue is defined as the inability to sustain the desired amount of productivity, categorized into muscular fatigue, metabolic fatigue, and mental fatigue [33]. Performing a strenuous work task for a short duration or performing a sub-maximal workload for a prolonged duration could cause undue fatigue, which acts as an internal perturbation to the postural control system [8]. Such internal perturbations challenge the individual’s upright maintenance, which manifests as an increased postural sway leading to loss of balance. Upon an inability to recover from such imbalance, a fall occurs [6]. During load carriage tasks, muscle fatigue has been commonly observed [22,23]. External loads have been shown to cause fatigue in the trunk, hip, and ankle musculature [34]. This muscular fatigue impairs the force production of the muscle over time. It is suggested that the fatigued muscles result in diminished proprioception by the muscle spindles and Golgi tendon organs [35]. These affected receptors impair the somatosensory feedback to the postural control system causing balance decrements. This impact on the afferent system manifests as higher muscle activity, indicating greater muscle activity requirement for postural control with fatigue [36]. Thus, the balance decrements observed in the study could be attributed to the undue fatigue caused by anterior load carrying and prolonged walking.

The majority of the previous studies conducted to observe the impact of fatigue on postural stability have used a larger workload over a shorter time as the fatiguing protocol [8,21]. A limited number of investigators have used a sub-maximal workload for prolonged exposure to induce fatigue and the results of the current study are consistent with their findings [13,20]. Wade and Davis exposed a group of young individuals up to two hours of simulated a roofing task on an inclined dry surface and observed greater balance decrements following the workload. Similarly, Chander et al. investigated increased postural sway over time following a four-hour walking session on a flat, dry surface [13].

### 4.2. Nature of the Walking Surface on Postural Stability 

In the current study, greater balance decrements on the slippery surface were observed in the unstable condition in the anteroposterior direction (COP-X average). This could be attributed to less friction between the shoe sole and the standing surface, which constantly challenges the postural control system’s ability to maintain an erect stance [37]. Human postural control is maintained through the communication between the afferent system (provided by the visual, vestibular, and somatosensory systems), central nervous system, and the efferent system. Altered, deranged, or ineffective communication between these systems would result in a loss of balance [9]. A lower amount of friction between the slippery surface and the participants’ shoes causes constant perturbations, requiring the postural control system to continuously correct the participants’ posture. Moreover, it is shown that the individuals acquire certain adjustments to the spatiotemporal parameters of gait during anticipated or encountered slips [33,36]. Thus, the balance decrements on the slippery surface could have occurred due to the anticipatory or reactive gait modifications that were acquired to minimize slipping [38]. However, gait was not studied in the current study, and further research is required to assess the gait modifications, possibly leading to postural instability. 

In the field of biomechanics, there is a dearth of literature regarding slip biomechanics. A limited number of studies were conducted on the impact of acute exposure to a slippery surface and comparison between dry and slippery surfaces regarding static balance. However, a few studies were done on acute exposure to a slippery surface and dynamic balance. According to the authors’ best knowledge, no previous studies were done on prolonged exposure to the slippery surface and static balance. Considering the comparison between dry and slippery surfaces, Yamaguchi et al. compared walking and turning on dry and slippery surfaces and observed similar findings to the present study. Their participants demonstrated greater balance decrements on the slippery surface compared to the dry surface [39]. Similarly, in a study conducted by Chander et al., greater balance decrements and slip propensity were observed on the slippery surface compared to the dry surface [14]. However, in the current study, the observed surface differences were limited to one postural sway COP parameter (COP in the anteroposterior direction) in the unstable condition. Thus, further investigation of the impact of the surface on static postural stability is required to make strong conclusions.

A time × surface interaction was observed in the stable condition in the anteroposterior direction (COP-X average). COP-X at 60 min was significantly lower for the slippery surface compared to 0 min and 30 min. Further, COP-X for the slippery surface was significantly lower compared to the dry surface at 60 min. However, these findings of the interaction were contrary to our original hypothesis, in that better postural stability was observed in the slippery surface compared to the dry surface at 60 min of exposure, and for the slippery surface, greater postural stability was observed at 60 min compared to 0 min and 30 min. This may be attributed to anticipatory postural responses, where individuals anticipated a greater need for maintaining postural stability when exposed to 60 min of slippery surface and may have compensated with a greater co-contraction of postural muscles, resulting in more medial-lateral stability. However, this is the only interaction in the analysis contrary to the original hypothesis and needs to be interpreted with caution. Further studies would aid in investigating the impact of dry and slippery surfaces on static postural stability.

There were some limitations to the study. The study was performed on a young, healthy population with a 10% anterior load carrying. Results could be different in geriatric and pathological populations. Only males were selected due to the availability of slip-resistant footwear. Moreover, the observed surface differences were only limited to one sway parameter in the unstable condition. This was a major limitation of the present study, requiring further investigation. Future studies could be directed towards different populations, females, different quantities, and different load-carrying methods such as posterior or lateral load carrying. Participants’ gait could be analyzed to assess the association between possible gait modifications and greater balance decrements on the slippery surface.

## 5. Conclusions

The study demonstrated significantly greater balance decrements with increasing walking time. This finding indicates that fatigue is unavoidable during work-related tasks, especially during prolonged tasks, hence the importance of rest-time scheduling in occupational settings. Additionally, the results are favorable of greater balance decrements on the slippery surface. However, further studies are required to investigate the comparison between the impact of dry and slippery surfaces on static postural stability. The study would contribute to the dearth of literature in slip biomechanics with prolonged exposure to a slippery surface and anterior load carrying. 

## Figures and Tables

**Table 1:** Descriptive statistics of the postural sway variables at 0 min, 30 min, and 60 min for dry and slippery surfaces.

Sway Parameter	Dry Surface			Slippery Surface		
	0 min	30 min	60 min	0 min	30 min	60 min
*Stable condition*						
COP-X average	1.34 ± 0.80	1.42 ± 0.81	1.65 ± 1.05	1.43 ± 0.84	1.49 ± 0.78	1.04 ± 0.57
COP-Y average	4.57 ± 2.12	4.76 ± 1.82	4.59 ± 3.20	5.00 ± 1.82	4.21 ± 1.46	4.30 ± 1.77
Average displacement along *X* axis	0.31 ± 0.12	0.41 ± 0.15 *	0.41 ± 0.19	0.35 ± 0.15	0.41 ± 0.18 *	0.44 ± 0.26
Average displacement along *Y*-axis	0.41 ± 0.09	0.55 ± 0.22 *	0.55 ± 0.19 *	0.43 ± 0.19	0.57 ± 0.19 *	0.55 ± 0.31 *
95% ellipsoid area	6.24 ± 4.47	13.13 ± 12.72 *	12.03 ± 8.92 *	9.54 ± 8.01	10.47 ± 6.71 *	11.62 ± 10.68 *
*Unstable condition*						
COP-X average	0.78 ± 0.49	1.09 ± 0.78	1.03 ± 0.62	1.05 ± 0.71 #	1.34 ± 0.81 #	1.13 ± 0.71 #
COP-Y average	2.74 ± 1.98	3.04 ± 2.27	3.02 ± 2.16	3.01 ± 1.51	2.96 ± 1.73	2.86 ± 1.35
Average displacement along *X* axis	0.47 ± 0.16	0.54 ± 0.18	0.54 ± 0.19	0.48 ± 0.17	0.46 ± 0.16	0.49 ± 0.13
Average displacement along *Y*-axis	0.60 ± 0.23	0.66 ± 0.19	0.72 ± 0.28	0.60 ± 0.16	0.65 ± 0.21	0.63 ± 0.22
95% ellipsoid area	11.54 ± 8.06	15.34 ± 8.07	15.35 ± 9.21	11.85 ± 8.31	12.98 ± 9.08	12.06 ± 7.32

Values are reported as mean ± standard deviation (SD). * denotes significant time differences compared to 0 min. # denotes significant surface differences compared to dry surface at *p* < 0.05.

**Table 2:** ANOVA table for different times (0 min, 30 min, and 60 min), surfaces (dry and slippery) & time × surface interaction.

Sway Parameter	Time			Surface			Time*Surface		
*Stable condition*	*F*	*p*	*η2*	*F*	*p*	*η2*	*F*	*p*	*ηp2*
COP-X average	0.48	0.626	0.03	1.39	0.254	0.08	4.46	0.019 *	0.21
COP-Y average	0.88	0.423	0.05	0.10	0.751	0.01	1.24	0.301	0.07
Average displacement along *X* axis	4.55	0.018 *	0.21	0.18	0.681	0.01	0.29	0.753	0.02
Average displacement along *Y*-axis	11.44	0.000 *	0.40	0.19	0.667	0.01	0.07	0.932	0.00
95% ellipsoid area	5.42	0.009 *	0.24	0.00	0.977	0.00	2.26	0.120	0.12
*Unstable condition*									
COP-X average	2.33	0.113	0.12	4.50	0.049 *	0.21	0.16	0.852	0.01
COP-Y average	0.10	0.908	0.01	0.00	0.978	0.00	0.36	0.697	0.02
Average displacement along *X* axis	0.99	0.382	0.06	0.86	0.366	0.05	1.18	0.320	0.07
Average displacement along *Y*-axis	2.68	0.083	0.14	1.10	0.310	0.06	1.21	0.310	0.07
95% ellipsoid area	1.94	0.159	0.10	0.61	0.447	0.03	1.20	0.315	0.07

* denotes significant differences between times, surfaces, or time × surface interaction at *p* < 0.05.
